# Assessing Physiotherapists’ Adherence to Clinical Practice Guidelines for Ankle Sprain Management in Saudi Arabia: A Cross-Sectional Study with National Online Survey

**DOI:** 10.3390/jcm14061889

**Published:** 2025-03-11

**Authors:** Abdulaziz Matouk Althumali, Hosam Alzahrani

**Affiliations:** Department of Physical Therapy, College of Applied Medical Sciences, Taif University, P.O. Box 11099, Taif 21994, Saudi Arabia; abdulazizalthumali@hotmail.com

**Keywords:** adherence, attitudes, clinical practice guidelines, ankle sprain management, physiotherapy, rehabilitation

## Abstract

**Background/Objectives:** Ankle sprain is one of the most common sports injuries globally. Despite its prevalence, the adequacy of knowledge in managing ankle sprain among physiotherapists in Saudi Arabia has not been assessed. This study aimed to assess the knowledge and degree of adherence to clinical practice guidelines (CPG) for the management of ankle sprains among physiotherapists. **Methods:** This study was a cross-sectional with national online questionnaire administered to participants through an online platform. It comprised three sections. The first section collected demographic data. The second section presented two clinical cases as the basis for the participants’ management decisions (the first with negative Ottawa Ankle Rules (OAR) and the second with positive OAR); participants were classified as “following”, “partially following”, “not following”, and “partially not following” the CPGs. In the third section, on a Likert scale (1–5), participants indicated how much they agreed with various CPGs statements. **Results:** A total of 381 physiotherapists (mean age: 28 ± 5; male: 57.1%) completed the questionnaire. In the case of acute ankle sprain with negative OAR, 0.2% of the participants were considered as “following” CPGs, 31.4% as “partially following”, 19.6% as “partially not following”, and 48.5% as “not-following”. In the case of acute ankle sprain with positive OAR, 5.2% were considered as “following” CPGs, 55.9% as “partially not following”, and 38.8% as “not following”. The knowledge assessment section elicited a 50% agreement among the participants on the 11 provided statements. **Conclusions:** Most physiotherapists have suboptimal adherence to CPG for managing ankle sprains, thus highlighting an evidence-to-practice gap.

## 1. Introduction

Ankle sprains are among the most widespread musculoskeletal injuries, impacting both general and sports populations [1]. Worldwide, millions of people are impacted by ankle sprain annually, resulting in substantial healthcare utilization and long-term functional impairments [2]. In Saudi Arabia particularly, the prevalence of ankle sprain ranges from 63.8% up to 77.7% [3,4]. Although they are often regarded as minor injuries that require minimal treatment and can heal quickly [2,5], ankle sprains can cause chronic ankle instability (CAI) [6], post-traumatic ankle osteoarthritis [7], and an increased risk of future falls [8]. CAI is a condition where a person’s ankle joint is mechanically and functionally unstable, characterised by issues like “giving way” or feelings of instability that persist for at least a year after the initial sprain [9]. Individuals with CAI typically require long-term management by healthcare providers or physiotherapists, which can take years [2].

Physiotherapists play a crucial role in providing evidence-based practice (EBP) management for patients with musculoskeletal diseases, including ankle sprains [10,11]. To promote the utilisation of EBP in ankle sprain management, various organisations, such as the American Physical Therapy Association, Academic Medical Center in Amsterdam, and the National Athletic Trainers’ Association, have developed consensus statements for the diagnosis, assessment, and treatment of ankle sprains [12,13,14,15,16]. When managing a patient with an ankle sprain, therapists should analyse risk factors such as previous ankle sprains, type and level of sports participation, workload, and deficiencies in proprioception and range of motion. They should also evaluate risk factors for developing instability such as lack of proprioception or balance exercises following an acute ankle sprain [12,14,15]. Clinicians can incorporate outcome measures in their assessment to identify the presence and severity of ankle instability using reliable and valid tools such as the Cumberland Ankle Instability Tool and evaluate functionality with validated patient-reported outcome measures such as the Foot and Ankle Ability Measure [17]. The Ottawa Ankle Rules (OAR), another established questionnaire and research protocol, should be used to determine whether radiography is required to rule out ankle or foot fractures [18]. Special tests, such as the Reverse Anterolateral Drawer Test, are valuable for evaluating ligamentous integrity [19]. However, existing guidelines provide evidence that the severity of ligament injury can be evaluated most reliably by delayed physical examination (4–5 days after trauma) [12,13].

Recommended therapeutic interventions for acute ankle sprains include the use of external support, such as braces or taping, and gradual weight-bearing on the affected limb. This can help alleviate pressure, promote healing, and reduce pain and swelling. Additionally, manual therapy combined with therapeutic exercises can improve ankle and foot mobility, reduce swelling, and enhance gait parameters [12]. Cryotherapy, which involves intermittent ice application and a therapeutic exercise program, can alleviate symptoms and improve function after acute ankle sprain [12,13]. While electrotherapy shows mixed results [12], low-level laser therapy can be employed for pain reduction during the initial phase [12]. However, ultrasound should not be used to manage acute ankle sprains [12,13,14]. The therapeutic effect of acupuncture remains inconclusive, while local vibration therapy shows promise but requires further research [13].

Despite the development of clinical practice guidelines (CPGs), their implementation is not guaranteed [20,21]. Recent evidence found an increasing trend of non-adherence to CPGs among healthcare professionals [22,23,24]. This discrepancy between recommended guidelines and actual clinical practice is commonly referred to as the “evidence-to-practice gap”. Numerous studies have investigated the use of EBP guidelines for various musculoskeletal disorders across different countries [24], but only a few have specifically investigated clinician adherence to EBP guidelines for the management of ankle sprains [25,26,27,28].

Research studies have consistently shown the benefits of EBP in managing musculoskeletal disorders. High adherence to EBP among physiotherapists leads to improved patient outcomes across a range of musculoskeletal disorders [28,29,30,31]. However, research from Saudi Arabia demonstrates a concerning gap in EBP application. One study demonstrated that most Saudi physiotherapists had no formal training in EBP [32], while another found that almost half of the practitioners held negative or neutral attitudes toward integrating EBP guidelines into their practice [33]. Additional research has underscored challenges such as a lack of understanding of musculoskeletal standards and insufficient expertise, confidence, and awareness in managing musculoskeletal conditions such as low back pain [34,35,36].

Limited research has been conducted in various countries, including Saudi Arabia, to investigate the extent to which physiotherapists adhere to EBP guidelines for specific musculoskeletal disorders. Furthermore, no study has specifically addressed the adherence of physiotherapists to EBP guidelines for ankle sprain management in Saudi Arabia. Consequently, it remains unclear whether physiotherapists in Saudi Arabia are aware of and adhere to EBP guidelines and recommendations for ankle sprain management. Therefore, this study aimed to assess the knowledge of and adherence to EBP recommendations and guidelines for ankle sprain management among physiotherapists in Saudi Arabia.

## 2. Materials and Methods

### 2.1. Study Design

This was a descriptive and cross-sectional study. Ethical approval for the study protocol was obtained from the Research Ethics Committee at Taif University, Taif, Saudi Arabia (No. 44-096). To ensure appropriate reporting, we followed the Strengthening the Reporting of Observational Studies in Epidemiology (STROBE) guidelines [37].

### 2.2. Participants and Sample Size

This study included physiotherapists with a valid professional licence issued by the Saudi Commission for Health Specialties (SCFHS) who are actively working in various healthcare facilities throughout Saudi Arabia, including clinics, hospitals, and rehabilitation facilities. Eligible participants had to have treated at least two patients with ankle sprains in the previous 12 months, be willing to fill out the questionnaire, and give informed consent. This criterion was determined based on preliminary observations of local referral patterns, which indicate that many physiotherapists encounter ankle sprain cases infrequently. By requiring a minimum of two cases, we ensured that the inclusion of physiotherapists with some degree of relevant clinical experience while still maintaining a representative sample of practitioners in Saudi Arabia.

Physiotherapists working outside Saudi Arabia, those in non-clinical settings (e.g., academic settings without direct patient care), and those with insufficient questionnaire answers were excluded. This strategy ensured that the study focused on physiotherapists who recently treated ankle sprains in various clinical settings.

The recruitment process involved distributing the questionnaire through various channels, including email and hospital visits, to eligible participants in various targeted clinics, hospitals, and rehabilitation facilities. This method of distributing the questionnaire was followed to ensure maximum participation of physiotherapists from different regions and practices throughout Saudi Arabia. The questionnaire was administered using Google Form. The required sample size was calculated using Cochran’s formula [38]. At the time of data collection, statistics from the SCFHS indicated that approximately 12,544 physiotherapists were practicing in Saudi Arabia. Based on a confidence level of 95% (Z = 1.96), margin of error of 5% (d = 0.05), and conservative proportion of 50% (p = 0.5), the minimum calculated sample size for this study was 373. The following formula was used for calculating the sample size: n = (N × Z^2^ × p × (1 − p))/(d^2^ × (N − 1) + Z^2^ × p × (1 − p)).

### 2.3. Data Collection

This study gathered data over a six-month period from February to July 2023. The questionnaire was segmented into three key sections: demographic, adherence, and knowledge assessments. The demographic section captured essential participant characteristics, including age, sex, nationality, highest academic qualification, area of physical therapy practice, years of professional experience, workplace setting, prior participation in ankle-related courses, and the number of ankle sprain patients treated per month.

The second section used two clinical vignettes to assess adherence (Supplementary Table S1). The first vignette depicted a relatively mild ankle injury without signs of fracture (negative OAR), while the second presented a more severe re-injury with potential bone involvement (positive OAR). Participants were asked to recommend first-week physiotherapy treatment options by selecting from a list of 19 management and assessment strategies tailored to the simulated cases. The clinical vignettes utilized in this study were adapted from a previous study conducted in collaboration with a panel of experts specialising in ankle sprain management [25]. These vignettes covered a spectrum of injury severities and patient demographics, ensuring comprehensive coverage of typical clinical presentations. Supplementary Table S2 offers additional insights and recommendations based on CPGs.

The third section aimed at assessing knowledge and required participants to rate their agreement with 11 statements on a Likert scale ranging from 1 (completely disagree) to 5 (completely agree) (Supplementary Table S3). The adherence and knowledge assessment sections were based on the latest CPGs for ankle sprains and stability [12,13].

### 2.4. Statistical Analysis

All data analyses were performed using the IBM SPSS Statistics software (version 23.0; IBM Corp., Armonk, NY, USA). Descriptive statistics were used to analyse demographic characteristics. Mean, standard deviation (SD), and frequency distributions were computed for each variable to provide a comprehensive overview of the cohort.

The participants’ responses to the two clinical scenarios were analysed using a percentage calculation to assess the degree of adherence to the EBP guidelines for ankle sprain management. The responses were categorised into four groups for each vignette: “following”, “partially following”, “not following”, and “partially not following”. The percentage of participants falling into each category was calculated based on their responses, as described below, providing an understanding of the overall adherence rates. Regarding the first vignette presenting a patient with negative OAR, participants’ responses were categorised as (1) “following” the recommendations if their selection strictly involved high-recommendation treatments (Level 1 or Grade A); (2) “partially following” if they opted for both high-recommendation treatments and those with lower recommendations (Level 2 or Grade B-C); (3) “partially not following” if they chose low-recommendation treatments (Level 2-3-4 or Grade C-D-E-F); and (4) “not following” if their interventions involved inappropriate treatments (e.g., ultrasound therapy, Grade A, etc.) either alone or alongside other treatments. Regarding the second vignette featuring a patient with positive OAR, the participants were classified as (1) “following” if the only selected action was to “consult a specialist or proceed to the emergency room”; (2) “partially not following” if they selected to “consult a specialist or proceed to the emergency room” in addition to the RICE protocol (Rest, Ice, Compression, Elevation), NSAID, referral to the doctor, and brace use only; and (3) “not following” if the decision either did not include specialist consultation or “proceed to the emergency room” or if treatment was initiated without excluding potential bone fractures.

Section 3, the knowledge assessment section, aimed to understand the level of agreement of the participants with statements retrieved from CPGs and recommendations. Responses on the 5-point Likert scale included 1 (completely agree), 2 (partially agree), 3 (neither agree nor disagree), 4 (partially disagree), and 5 (completely disagree). Consequently, responses 1 and 2 were considered as agreement, whereas response 3, 4, and 5 were regarded as disagreement with the EBP recommendations. To explore the perspectives of the participants on ankle sprain assessment and treatment, responses to the survey questions were qualitatively analysed using thematic analysis. Common themes and patterns were identified by systematically reviewing and coding the responses. These themes included the investigation of previous sprains, utilisation of functional outcome measures, long-term follow-up plans, and incorporation of modalities such as taping, bracing, and manual therapy techniques. The frequency of each theme was recorded to gauge the prevalence of specific perspectives within the sample.

## 3. Results

### 3.1. Participants Characteristics

In total, 381 participants were included, 57.1% were men, and the average age was 28.6 years (SD 4.8). Most participants had a bachelor’s degree (84.8%) and one to five years of clinical experience (41.5%). In terms of participation in a “rehabilitation of patients with an ankle sprain” course, 37.3% of participants reported attending at least one course. Table 1 presents the demographic characteristics of the participants.

### 3.2. Main Findings

Table 1 reveals important insights into the participants’ responses to vignettes 1 and 2. Regarding the first vignette, which described a case of acute ankle sprain without any signs of possible bone fracture, 48.6% of the participants were “not following” the guidelines. These participants recommended treatments that were not highly recommended (Grade A for not to be used). Additionally, 19.7% of the participants were “partially not following” the guidelines, suggesting only low-level recommended therapies (Level 2-3-4 or Grade C-D-E-F). Furthermore, 31.5% of the participants were “partially following” the guidelines, indicating that they chose a mix of highly recommended treatments (Grade A) alongside low-level recommended treatments (Grade C). Interestingly, only 0.3% of participants were “following” the guidelines, suggesting only the strongly advised medical interventions.

Regarding the second vignette, which represented a case of re-injury during the acute phase of an ankle sprain with positive symptoms and signs indicating a suspected bone fracture, the findings were concerning. We found that 38.8% and 55.9% of the participants were “not following” and “partially not following” the guidelines, respectively. Conversely, only 5.2% of the participants were “following” the guidelines.

**Figure 1:**
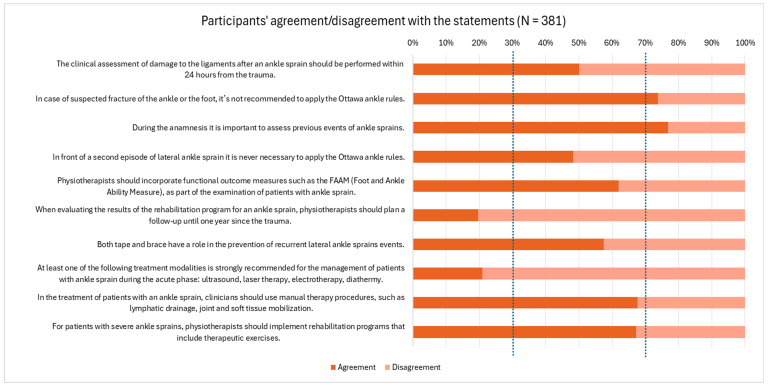
Participants’ Perspectives on Key Statements Regarding Ankle Sprain Assessment and Treatment.

Figure 1 provides important insights into participants’ perspectives on various statements related to ankle sprain assessment and treatment. There was consensus among the respondents regarding the importance of investigating the patient’s history of previous ankle sprains. Most participants agreed that the OAR should not be applied in cases of suspected ankle or foot fractures. However, the majority of participants disagreed with the notion that follow-up plans extending up to one year post trauma were necessary. Regarding treatment modalities, over 70% disagreed with the recommendation of using adjunct therapies, such as ultrasound, laser therapy, and diathermy, during the acute phase.

## 4. Discussion

This study aimed to investigate the knowledge of and degree of adherence to EBP guidelines for managing ankle sprains among physiotherapists in Saudi Arabia. Our findings indicate that the majority of Saudi physiotherapists appear to be unaware of and do not fully adhere to the EBP guidelines in their clinical practice. This contrasts with studies from other countries, which provide a somewhat different picture. For example, a study from the Netherlands found that majority of physiotherapists reported moderate compliance with ankle sprain guidelines, noting that factors such as systemic healthcare constraints, patient expectations, and unclear recommendations contributed to inconsistent adherence [27]. Similarly, research from Italy found that although physiotherapists were generally aware of evidence-based recommendations for ankle sprain management, a noticeable gap still existed between their knowledge and actual practice—with most physiotherapists incorporating lower-recommended treatments alongside first-line interventions [25]. In comparison to these international contexts, the level of unawareness and non-adherence among Saudi physiotherapists appears to be more pronounced. This difference highlights the critical need for targeted educational initiatives and broader system-level interventions to improve guideline awareness and implementation in Saudi Arabia.

The participants’ responses on ankle sprain assessment and treatment revealed important inconsistencies and variations when compared with the established guidelines and evidence-based recommendations. Most participants agreed on the need to promptly assess ligament damage within 24 h post injury. However, the preference for immediate ligament assessment differs from the recommendations, which suggest that the sensitivity and specificity of anterior talofibular ligament assessment are optimal when the clinical evaluation is delayed to between 4 and 5 days after injury. Furthermore, recent evidence suggests that delaying the evaluation to 4–5 days post injury enhances the diagnostic sensitivity and specificity of tests such as the anterior drawer test [39]. For example, in the acute setting, clinical signs such as lateral swelling demonstrate high sensitivity (100%), but the sensitivity and specificity of the anterior drawer test substantially improve in the delayed setting (e.g., the sensitivity increases from 21% to 61%) [39]. This demonstrates the trade-off between immediate intervention and the possibility of enhanced diagnostic accuracy with delayed assessments, which allow pain and swelling to resolve. In practice, while early assessment can effectively rule out or raise the suspicion of serious ligamentous injuries, a delayed approach may yield more reliable findings, emphasising the importance of adjusting the assessment timing to clinical conditions for optimal outcomes.

The application of the OAR also illustrates the variability in clinical practices. Most participants did not support the use of OAR for suspected fractures, diverging from the guideline recommendations that advocate its application to accurately confirm or rule out fractures and minimise unnecessary radiographic examinations. A recent meta-analysis reinforced the OAR as a cost-effective strategy for decreasing unnecessary radiographic referrals, thereby improving diagnostic efficiency and lowering medical costs [40]. This divergence suggests a gap in the alignment with evidence-based guidelines, demonstrating that some clinicians may not fully incorporate these recommendations into their clinical decision-making processes.

The treatment preferences we found reflect both adherence to and divergence from evidence-based guidelines. Most participants did not support adjunct therapies such as ultrasound, laser therapy, or diathermy during the acute phase. This is consistent with the guidelines that provide limited support for these modalities owing to the inconsistent evidence of their effectiveness, particularly discouraging the use of ultrasound and expressing moderate evidence against electrotherapy. The limited support for these therapies among therapists suggests an alignment with current research findings, showing awareness of the guidelines and a potential shift away from these modalities in clinical practice.

Moreover, there was strong support for the use of therapeutic exercises and manual therapy for the treatment of serious ankle sprains. This closely adheres to evidence-based guidelines that endorse these treatment methods for optimising function and reducing pain and the risk of recurrence. A recent meta-analysis demonstrated that the combination of manual therapy and therapeutic exercises was more effective than exercise alone in improving function, range of motion, and pain reduction in patients with lateral ankle sprains [41]. The alignment with rehabilitation-focused interventions demonstrated a commitment to structured therapeutic methods, reflecting a high level of adherence to best practices for severe cases. Overall, these findings suggest that while there is alignment with guidelines in several areas, particularly regarding preventive measures and rehabilitation approaches, there are opportunities for improved adherence regarding diagnostic and treatment modalities and follow-up duration.

Preventative measures also received robust endorsement, with participants favouring the use of both taping and bracing to decrease the risk of recurrent lateral ankle sprains. This practice adheres to the guideline recommendations that recognise bracing as an effective preventive approach, particularly for individuals with a history of ankle sprains. However, the follow-up plans diverged notably from the recommendations. Most participants showed a preference against extended follow-up for more than one year, in contrast to guidelines that recommend longer monitoring due to the high incidence of residual symptoms even a year post injury. This discrepancy may reflect clinical considerations regarding practicality and patient compliance, potentially balancing the ideal follow-up duration with feasible practice.

A lack of knowledge regarding musculoskeletal guidelines was previously identified as a substantial barrier among practitioners [34]. A previous study conducted in Saudi Arabia investigated physiotherapists’ behaviour, attitudes, awareness, and knowledge of EBP, showing a significant gap between the knowledge and application of EBP guidelines, as most practising physiotherapists reported no formal training in EBP [32]. Another study demonstrated that approximately half of the physiotherapists in Saudi Arabia held a negative or neutral attitude toward integrating EBP guidelines into their clinical practice [33], demonstrating challenges in comprehending and applying these recommendations. Additional research [35,36] revealed a lack of knowledge, confidence, and awareness among physiotherapists in Saudi Arabia regarding the implementation of EBP in cases such as low back pain and fibromyalgia. In contrast, studies from other countries such as Canada [31], India [29], the Netherlands [28], and Australia [30] have reported higher adherence rates to EBP guidelines among physiotherapists for various musculoskeletal disorders, underscoring regional differences in EBP comprehension and application.

The significance of this study cannot be understated because it underlines the urgent need for institutional mandates and robust support systems that prioritise and facilitate the implementation of evidence-based practice guidelines. It is imperative to incorporate the assessment of research knowledge as an essential criterion for promoting and fostering institutional support to encourage the integration of CPGs. By bridging the perceived gap between research and practice and promoting the adoption of CPGs among physiotherapists in Saudi Arabia we can significantly improve adherence to guidelines and enhance the quality of care.

One limitation of the current study is that it relied on self-reported data from an online questionnaire, which might have introduced selection bias. Another limitation is that this study recruited participants through a non-random convenience sampling approach. As access to physiotherapists’ information in Saudi Arabia was not possible, a convenience sampling technique was adopted. This may have limited the generalisability of the findings. Moreover, due to the use of Google Form for distributing the questionnaire, we were unable to detect the number of participants who received the survey invitation, which precludes calculation of response rate. Finally, the questionnaire used in the current study was adapted from a previous study without additional expert review or pilot testing in our context, which may impact the reliability and validity of the findings. These limitations should be considered when interpreting the results of this study.

## 5. Conclusions

This study found that physiotherapists in Saudi Arabia adhere sub-optimally to evidence-based guidelines for managing ankle sprains. To improve adherence to EBP, there should be integration and implementation of guidelines, support for continuing education opportunities, and effective monitoring and evaluation mechanisms. Implementing these measures could lead to optimal care delivery and improved patient outcomes.

## Figures and Tables

**Table 1:** Participant profiles by level of adherence.

		Clinical Vignette 1				Clinical Vignette 2		
	All Participants (N = 381)	“Not Following” (N = 185)	“Partially Not Following” (N = 75)	“Partially Following” (N = 120)	“Following” (N = 1)	“Not Following” (N = 148)	“Partially Not Following” (N = 213)	“Following” (N = 20)
Characteristics								
Age (years (mean (SD))		28.6 (4.8)	28.3 (4.7)	28.5 (5.8)	NA	28.5 (5.2)	28.5 (5.0)	28.9 (5.0)
Sex (N (%))								
Male	217 (57.1)	107 (57.8)	41 (54.7)	68 (56.7)	1 (100.0)	96 (64.9)	110 (51.6)	11 (55.0)
Female	163 (42.9)	78 (42.2)	34 (45.3)	52 (43.3)	0 (0.0)	52 (35.1)	103 (48.4)	9 (45.0)
Highest Academic Education Qualification (N (%))								
Diploma	10 (2.6)	7 (3.8)	1 (1.3)	2 (1.7)	0 (0.0)	5 (3.4)	5 (2.3)	0 (0.0)
Bachelor’s degree	322 (84.8)	163 (88.1)	56 (74.7)	103 (85.8)	1 (100.0)	123 (83.1)	182 (85.4)	18 (90.0)
Master’s degree	41 (10.8)	13 (7.0)	15 (20.0)	13 (10.8)	0 (0.0)	19 (12.8)	21 (9.9)	1 (5.0)
Ph.D.	7 (1.8)	2 (1.1)	3 (4.0)	2 (1.7)	0 (0.0)	1 (0.7)	5 (2.3)	1 (5.0)
Years of practice (N (%))								
Less than 1 year	108 (28.3)	51 (27.6)	19 (25.3)	37 (30.8)	1 (100.0)	47 (31.8)	58 (27.2)	3 (15.0)
From 1 to 5 years	158 (41.5)	78 (42.2)	28 (37.3)	52 (43.3)	0 (0.0)	57 (38.5)	90 (42.3)	11 (55.0)
From 6 to 10 years	74 (19.4)	38 (20.5)	22 (29.3)	14 (11.7)	0 (0.0)	26 (17.6)	44 (20.7)	4 (20.0)
More than 10 years	41 (10.7)	18 (9.7)	6 (8.0)	17 (14.2)	0 (0.0)	18 (12.2)	21 (9.8)	2 (10.0)
Participation in a course on “Rehabilitation of patients with ankle sprains” (N (%))								
Yes	142 (37.3)	59 (31.9)	34 (45.3)	49 (40.8)	0 (0.0)	72 (48.6)	65 (30.5)	5 (25.0)
No	239 (62.7)	126 (68.1)	41 (54.7)	71 (59.2)	1 (100.0)	76 (51.4)	148 (69.5)	15 (75.0)

Abbreviations: N, number.

## Data Availability

Data are available upon reasonable request from corresponding author.

## Supplementary Materials

The following supporting information can be downloaded at https://www.mdpi.com/article/10.3390/jcm14061889/s1. Table S1: Clinical vignettes; Table S2: Evidence-based practice recommendations. Table S3: Statements consensus–knowledge investigation.

## Abbreviations

The following abbreviations are used in this manuscript:

CAI	Chronic ankle instability
CPGs	Clinical practice guidelines
EBP	Evidence-based practice
N	Number
OAR	Ottawa Ankle Rules
RICE	Rest, Ice, Compression, Elevation
SD	Standard deviation
STROBE	Strengthening the Reporting of Observational Studies in Epidemiology
